# Proteomic analysis of broccoli (*Brassica oleracea*) under high temperature and waterlogging stresses

**DOI:** 10.1186/s40529-015-0098-2

**Published:** 2015-07-15

**Authors:** Hsin-Hung Lin, Kuan-Hung Lin, Su-Ching Chen, Yu-Hsing Shen, Hsiao-Feng Lo

**Affiliations:** 1 Department of Horticulture and Landscape Architecture, National Taiwan University, Taipei, Taiwan; 2 Department of Horticulture and Biotechnology, Chinese Culture University, Taipei, Taiwan; 3 Institute of plant and microbial biology, Academia Sinica, Taipei, Taiwan

**Keywords:** Ribulose bisphosphate carboxylase, Broccoli, Heat and waterlogging stresses, Differentially expressed proteins

## Abstract

**Background:**

The production of broccoli (*Brassica oleracea*) is largely reduced by waterlogging and high temperature stresses. Heat-tolerant and heat-susceptible broccoli cultivars TSS-AVRDC-2 and B-75, respectively, were used for physiological and proteomic analyses. The objective of this study was to identify TSS-AVRDC-2 and B-75 proteins differentially regulated at different time periods in response to waterlogging at 40 °C for three days.

**Results:**

TSS-AVRDC-2 exhibited significantly higher chlorophyll content, lower stomatal conductance, and better H_2_O_2_ scavenging under stress in comparison to B-75. Two-dimensional liquid phase fractionation analyses revealed that Rubisco proteins in both varieties were regulated under stressing treatments, and that TSS-AVRDC-2 had higher levels of both Rubisco large and small subunit transcripts than B-75 when subjected to high temperature and/or waterlogging.

**Conclusions:**

This report utilizes physiological and proteomic approaches to discover changes in the protein expression profiles of broccoli in response to heat and waterlogging stresses. Higher levels of Rubisco proteins in TSS-AVRDC-2 could lead to increased carbon fixation efficiency to provide sufficient energy to enable stress tolerance under waterlogging at 40 °C.

**Electronic supplementary material:**

The online version of this article (doi:10.1186/s40529-015-0098-2) contains supplementary material, which is available to authorized users.

## Background

Broccoli (*Brassica oleracea var. italica*) is a member of the Brassicaceae family. This cole crop is not only economically important but also provides nutrients that have anticancer characteristics (Aggarwal and Ichikawa 2005). Under optimal temperatures of 18 to 25 °C, this plant grows normal flower buds and forms one large central head. However, when temperature exceeds 30 °C, heads become loose and branchy, tend to develop leaf-like structures, and initial floret development is disrupted (Bjorkman and Pearson 1998). Taiwan is located in tropical and subtropical regions where daily temperatures exceed 30 °C during summer (June to September), thus reducing the quality and quantity of broccoli produced. Moreover, typhoons always bring heavy rains in summer, and flooding from typhoon rainfall is a major risk to fresh-market broccoli production when the soil is water-saturated.

Previous studies report that when plants are subjected to high temperature and waterlogging, the water and chlorophyll in leaves are decreased but the level of hydrogen peroxide in the cells is increased, resulting in reduced plant growth and development (Moller and Kristensen 2004; Kumutha et al. 2009; Pucciariello et al. 2012). Fortunately, plants have developed several mechanisms to protect themselves from stressful abiotic environments, some of which overlap. For example, pretreating *Arabidopsis* with 38 °C for 90 min results in a higher tolerance to waterlogging (Banti et al. 2010). Additionally, waterlogging can also stimulate the expression of heat shock factors and proteins as effectively as high temperature (Banti et al. 2010). These findings demonstrate that responses to different stress factors in plants are coordinated by complex and interconnected signaling pathways. Different sets of genes have been identified that activate against various abiotic stresses, including heat, flood, drought, cold, and intense light (Kreps et al. 2002; Seki et al. 2002; Rizhsky et al. 2004). However, little is known about how the simultaneous occurrence of multiple abiotic stresses, as opposed to individual stresses, damage crop production (Rizhsky et al. 2004; Mittler 2006; Atkinson and Urwin 2012). Therefore, uncovering the physiological mechanisms whereby plants can withstand combined waterlogging and high temperature stressing is greatly desired.

Proteomic analysis is a powerful approach for revealing differentially expressed proteins under given conditions. Liu et al. discovered a large number of differentially expressed proteins from broccoli florets treated with N^6^-benzylaminopurine, illuminating a complex network that provides comprehensive information on post-harvest yellowing response mechanisms (Liu et al., 2011; Liu et al., 2013). Using two-dimensional liquid phase fractionation (PF2D) and matrix-assisted laser desorption ionization time-of-flight mass spectrometry (MALDI-TOF MS), we identified 31 differentially expressed proteins from heat-tolerant and heat-susceptible broccoli cultivars TSS-AVRDC-2 and B-75, respectively, under high temperature and/or waterlogging stresses. We then further cloned the stress-responsive Rubisco genes and their transcript levels under stressing were determined. The possible mechanism whereby TSS-AVRDC-2 broccoli develops florets during summer is also discussed herein.

## Methods

### Plant materials, culturing, and heat- and flood-stress treatments

Seeds of broccoli (*B. oleracea*) varieties TSS-AVRDC-2 and B-75 were obtained as gifts from Mr. L.C. Chung (see Acknowledgments) at AVRDC (Vegetable Research and Development Center) - the World Vegetable Center, Tainan, Taiwan. B-75 is a heat-sensitive variety that requires optimum growing temperatures for satisfactory production. However, TSS-AVRDC-2 is a heat-tolerant variety suitable especially for warm-subtropical regions such as southern Taiwan where average day temperatures reach as high as 40 °C during summer. Seeds of TSS-AVRDC-2 and B-75 were immersed in distilled-deionized (dd) water in darkness for 24 h and germinated on wetted Whatman filter papers for three days to ensure uniform germination. Seedlings were then transplanted into 5-inch plastic pots containing a mixture of peat and moss (4:1, v:v) and placed in a growth chamber under 300 μmol m^−2^ s^−1^ light with a 16 h photoperiod provided by fluorescent and incandescent light. The temperature was maintained at 22 °C in the light and 18 °C in the dark, with a relative humidity (RH) of 80 %. Plants were watered with a half-strength Hoagland solution (Hoagland and Arnon 1950) three times a week to maintain optimal irrigation and growth for 40 days before imposing heat and waterlogging stresses.

Pots of TSS-AVRDC-2 and B-75 plants were divided into four groups and transferred to 20 °C without waterlogging (C, control), 20 °C with waterlogging (F), 40 °C without waterlogging (H), and 40 °C with waterlogging (HF) for periods of 0, 12, 24, 48, and 72 h (for chlorophyll content and H_2_O_2_ content experiments) or 0, 24, 36, 48, 60, 72, 96, 120 and 144 h (for stomatal conductance experiment) in four growth chambers under the above-described conditions. In the waterlogging treatments, pots were randomly placed in 28 × 14 × 14 cm plastic buckets and subjected to flooding by filling the buckets with tap water to 5 cm above the soil surface. Pots were removed from the buckets at different times following flooding, and plants were removed and leaves from each plant were clipped, frozen in liquid nitrogen, and stored at −80 °C in an ultrafreezer until use. Three replicates from each time interval for the four treatments were randomly placed in a growth chamber. The experiment was performed twice independently for a randomized design of growth environment, sampling day, and physiological analysis.

### Measurement of chlorophyll content (CC), stomatal conductance (SC), and H_2_O_2_ content (HC)

Characteristic physiological responses of plants to different treatments were evaluated to determine if the defense mechanisms of the genotypes are related to their CC, SC, and HC values. Relative CC unit leaf area was determined according to a previous study (Davenport et al. 2005) using a SPAD (Soil Plant Analysis Development) analyzer (SPAD-502 Chlorophyll Meter, Konica Minolta, Tokyo, Japan). Stomatal conductance values were measured with a Porometer (Decagon Devices, Pullman, WA). The sensor was equilibrated by considering water vapor in the ambient atmosphere as 0 % relative humidity and wetted filter paper at 100 % relative humidity. After equilibration, each leaf was inserted into the head of the Porometer sensor and a measurement taken (Limm et al. 2009). HC measurement was previously described (Wei et al. 2012). Briefly, each leaf was homogenized in liquid nitrogen and ice-cold extraction buffer (50 mM sodium phosphate buffer, pH 6.8, and 1 mM hydroxylamine) then added. The mixture was centrifuged at 12,000 *g* for 10 min at 4 °C and an equal amount of titanium reagent (0.1 % TiCl_2_ in 20 % H_2_SO_4_) added to the supernatant. The titanium reagent mixtures were centrifuged at 12,000 *g* for 10 min and supernatants measured at 410 nm absorbance. H_2_O_2_ content was computed from a standard curve prepared from H_2_O_2_ solutions of known concentrations.

**Fig. 1:**
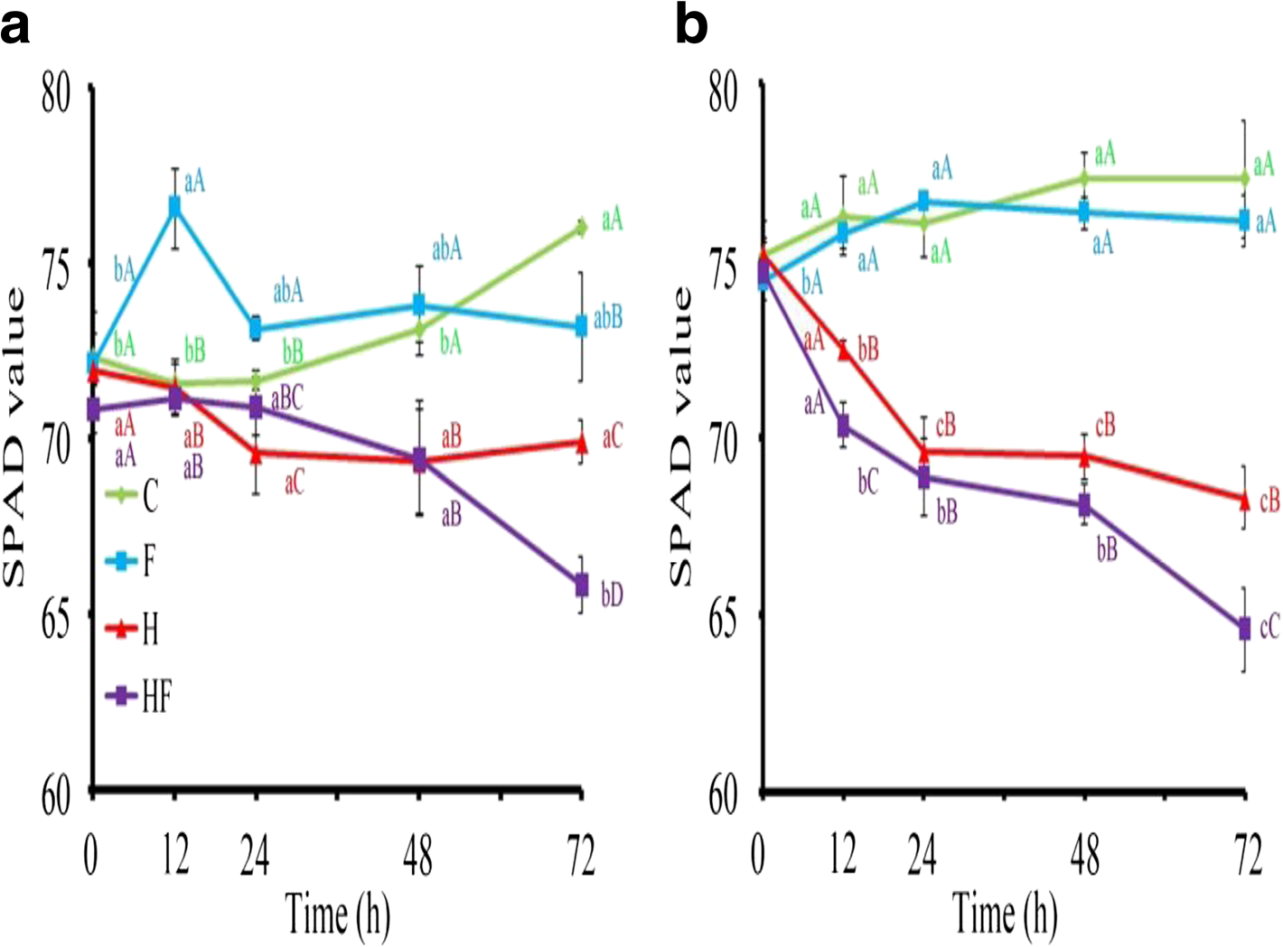
Chlorophyll content in stressed plants. Leaf SPAD values in TSS-AVRDC-2 (**a**) and B-75 (**b**) plants under control (C), waterlogging (F), 40 °C (H), and waterlogging at 40 °C (HF) treatments for 0, 12, 24, 48, and 72 h. Values represent the means of five independent plants. Values represent the means of five independent plants. Means with the same small letters are not significantly different among times within the same row, based on LSD at *p* ≤ 0.05 under ANOVA. Means with the same capital letters are not significantly different between treatments within the same time point, based on LSD at *p* ≤ 0.05 under ANOVA

**Fig. 2:**
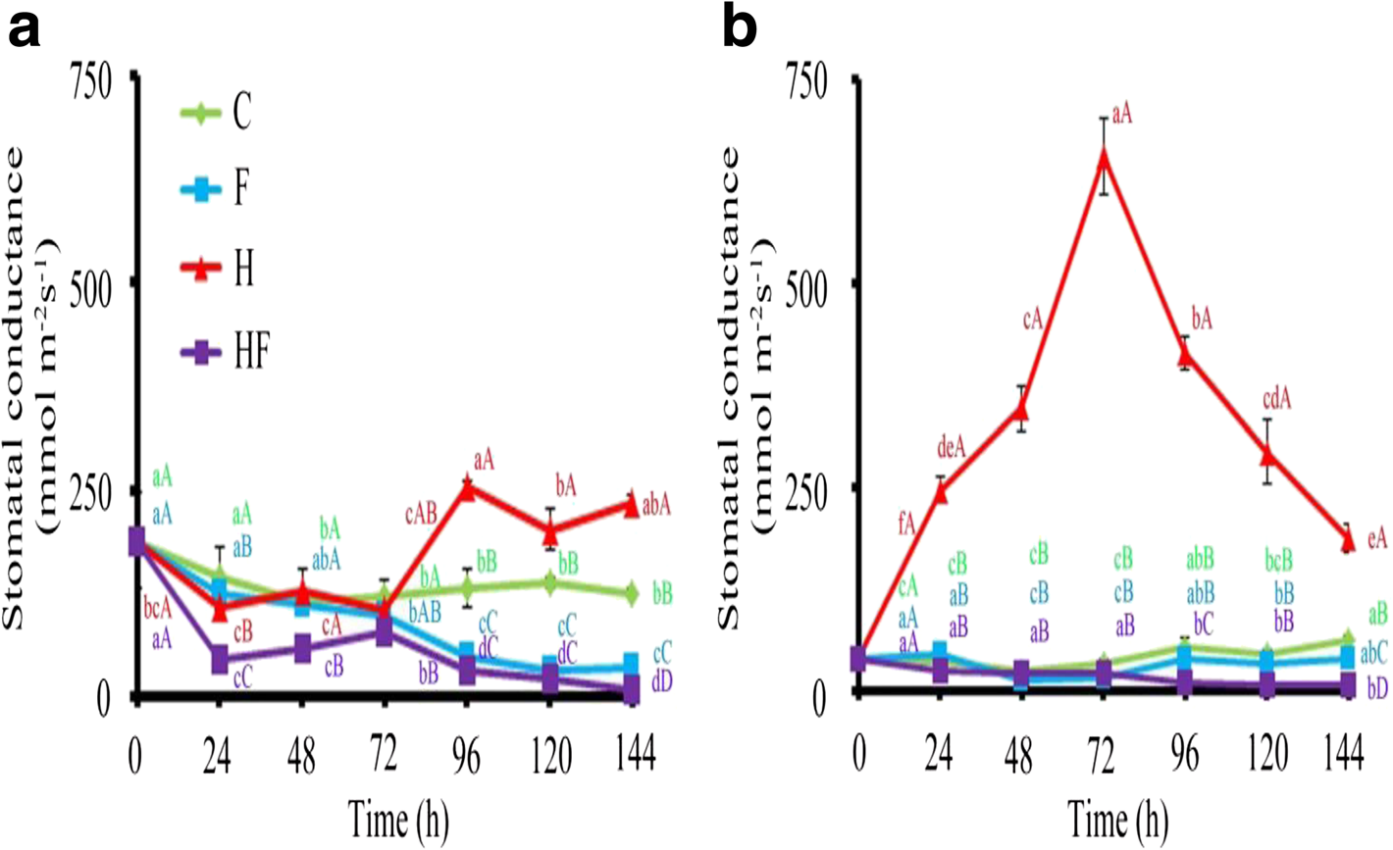
Stomatal conductance in stressed plants. Stomatal conductance in TSS-AVRDC-2 (**a**) and B-75 (**b**) plants under control (C), waterlogging (F), 40 °C (H), and waterlogging at 40 °C (HF) treatments for 0, 24, 72, 48, 96, 120, and 144 h. Values represent the means of five independent plants. Values represent the means of five independent plants. Means with the same small letters are not significantly different among times within the same row, based on LSD at *p* ≤ 0.05 under ANOVA. Means with the same capital letters are not significantly different between treatments within the same time point, based on LSD at *p* ≤ 0.05 under ANOVA

**Fig. 3:**
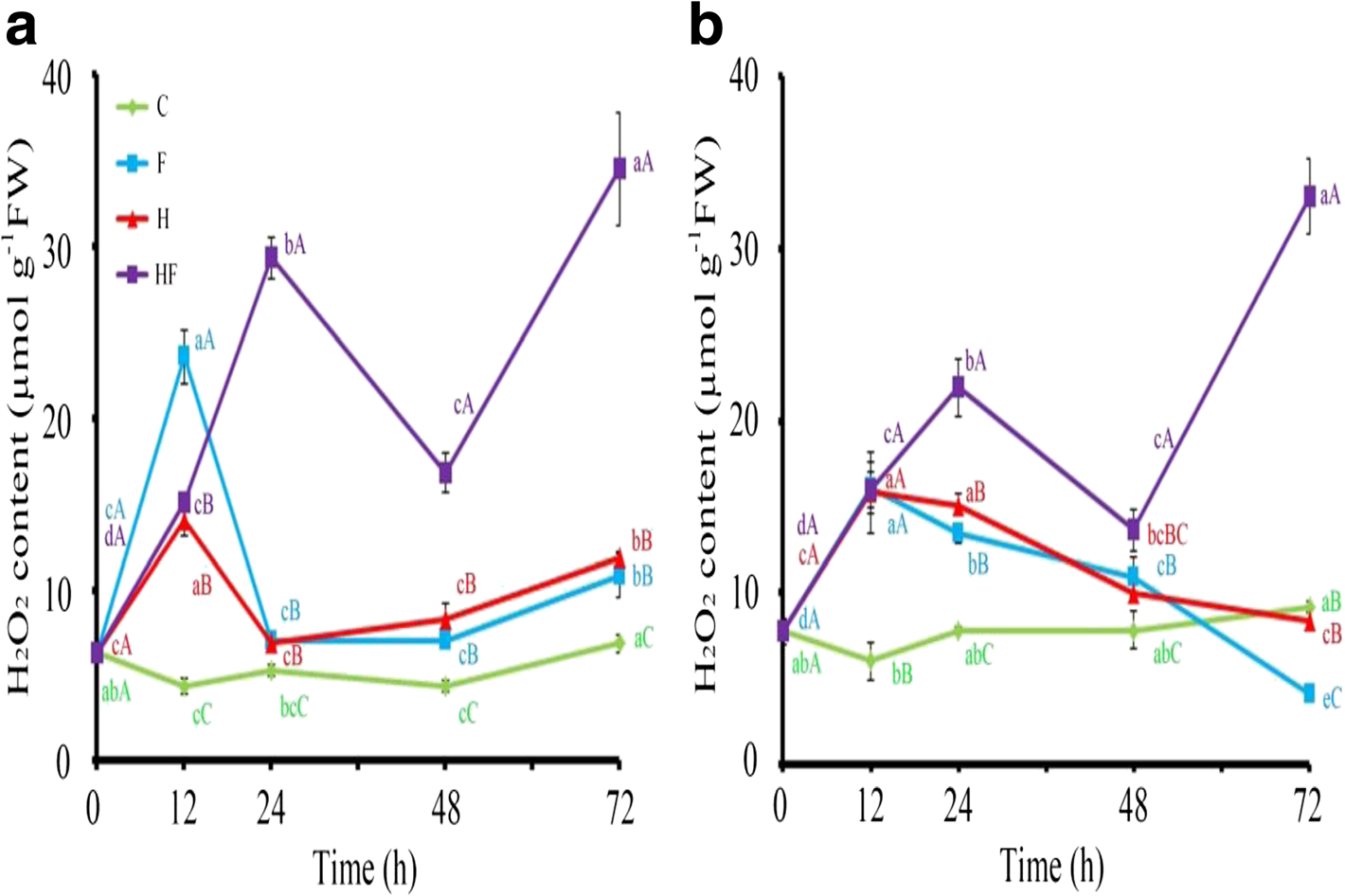
H_2_O_2_ content in stressed plants. Leaf H_2_O_2_ content in TSS-AVRDC-2 (**a**) and B-75 (**b**) plants under control (C), waterlogging (F), 40 °C (H), and waterlogging at 40 °C (HF) treatments for 0, 12, 24, 48, and 72 h. Values represent the means of five independent plants. Means with the same small letters are not significantly different among times within the same row, based on LSD at *p* ≤ 0.05 under ANOVA. Means with the same capital letters are not significantly different between treatments within the same time point, based on LSD at *p* ≤ 0.05 under ANOVA

Data shown in Figs. 1, 2 and 3 represent the mean of at least two independent sets of experiments with similar results. Measurements of physiological parameters were analyzed by analysis of variance (ANOVA) with completely randomized design. For significant values, means were separated by the least significant difference (LSD) test at *p* ≤ 0.05 using PC SAS 8.2 (SAS Institute, Cary, NC, USA).

### Protein isolation and quantification

Two grams of frozen leaf samples were ground into powder in liquid nitrogen, extracted with 7 ml extraction buffer (5 % [v/v] 2*-*mercaptoethanol*,* 2 % [w/v] polyvinylpolypyrrolidone, 0.5 % [w/v] sodium dodecyl sulfate, 10 % [v/v] glycerol, and 60 mM Tris–HCl, pH 6.8), and centrifuged for 30 min at 13,000 *g* at 4 °C. Supernatants were transferred to a new tube and ice-cold acetone was added, and placed at −20 °C for 1 h. Precipitated proteins were collected after centrifuging at 5,000 *g* for 30 min at 4 °C. Pellets were then washed with ice-cold acetone three times, vacuum-dried, and kept at −80 °C until use. Protein concentration was determined using the Bradford method (Protein Assay, Bio-Rad Laboratories, Hercules, CA), and bovine serum albumin (BSA) was used as a protein standard.

### 2-D liquid phase fractionation analysis and protein identification by MALDI-TOF MS and database search

A first-dimension high performance chromatofocusing column (Beckman Coulter, CA, USA) was pre-equilibrated with starting buffer (20 mM Tris–HCl, pH 8.5) until column pH reached 8.3. Samples were then injected into the column at a flow rate of 0.2 ml/min and column effluent was monitored at an absorbance of 280 nm. The pH gradient was created by an elution buffer (polybuffer 74, pH 4.0; GE Healthcare, NJ, USA) and fractions were collected every 0.3 pH unit. In the second dimension, fraction separation was performed using a 4.6 x 30 mm nonporous C18 HPRP column (Beckman Coulter) at 50 °C at a flow rate of 0.75 ml/min. Solvent A (0.1 % w/v trifluroracetic acid) and solvent B (0.08 % w/v acetonitrile) were used to create a gradient. The gradient consisted of solvent A at 100 % for 10 min and solvent B at 0 to 60 % for 30 min, and eluent was collected every 15 s, and was monitored at an absorbance of 214 nm. Protein peaks from those treated and un-treated plants were compared using DeltaVue software (Beckman Coulter, Inc. Fullerton, CA, USA). DeltaVue allowed side-by-side viewing of the second-dimension runs for two samples, and was used to compare and quantify the number of differential expressed protein between them. Each band shown in this chromatogram represented a singly separated protein and the relative intensity of the colors was directly proportional to the difference in protein concentration. The up-regulation and down-regulation of the proteins were identified (Additional file 1: Figures S1 and Additional file 2: Figure S2).

The eluents were dried under a vacuum and the pellet dissolved in a reducing solution (50 mM ammonium bicarbonate and 10 mM dithiothreitol) for 60 min at 60 °C. After reduction steps, protein solutions were digested at 37 °C for 16 h with 5 ng/μL of trypsin. Tryptic digestion was stopped by adding 1 % (v/v) formic acid. Digested proteins were further analyzed by MALDI-TOF MS. Mass spectra values were searched against NCBI and Swiss-Prot protein databases using the Mascot search program (http://www.matrixscience.com). The search parameters were set as follows: 1 missed cleavage, fixed modification, peptide charge ±1, and variable modifications were carbamidomethyl of cysteine and oxidation of methionine. Trypsin was specified as the proteolytic enzyme. Peptide tolerance and MS mass tolerance were 50 ppm and 0.25 Da, respectively. Peptide mass fingerprinting match confidence was based on the MOWSE score and confirmed by accurate overlapping of matched peptides with mass spectrum major peaks. Scores greater than 67 (*p* < 0.05) were considered positive. Peaks with multiple proteins detected by MS were not considered. Only significant hits, as defined by MASCOT probability analysis (*p* < 0.05), were accepted. To identify differentially expressed proteins, the protein peaks from waterlogging (F), 40 °C (H), and 40 °C with waterlogging (HF) at 12-h and 24-h time points were compared to those from the control sample (20 °C).

### Amplification and cloning of RubS and RubL from TSS-AVRDC-2 and B-75

For amplification and cloning experiments, genomic (g) DNA was isolated from mature TSS-AVRDC-2 and B-75 leaves based on the CTAB-based method from a previous study (Han et al. 2012). In brief, each leaf sample was ground into powder in liquid nitrogen, extracted with 65 °C preheated CTAB extraction buffer (2 % [v/v] CTAB*,* 2 % [w/v] polyvinylpolypyrrolidone, 2 % [w/v] PVP-40000, 1.4 M NaCl, 2 % 2-mercaptoethanol, 100 mM Tris HCl, pH 8.0 and 20 mM EDTA, pH 8.0), incubated at 65 °C for 1 h, and centrifuged for 20 min at 13,000 *g* at 4 °C. An equal volume of chloroform was added to the supernatant. The supernatant was transferred and precipitated with isopropanol. Total RNA was isolated from leaves of TSS-AVRDC-2 and B-75 with Trizol reagent (Invitrogen, CA, USA) according to manufacturer instructions. In brief, each leaf sample was ground into powder in liquid nitrogen and extracted with Trizol reagent. Another 0.2 volume of chloroform was added and centrifuged for 15 min at 13,000 *g* at 4 °C. A 0.5 volume of isopropanol was added to the supernatant to precipitate RNA at 4 ° C for 2 h and centrifuged for 20 min at 13,000 *g* at 4 °C. The pellet was washed with 75 % ethanol twice and re-suspended in diethyl pyrocarbonate-treated deionized water. The quality of the total RNA was resolved by formaldehyde gel. Complementary (c) DNA was synthesized by using 1 ug of total RNA, Moloney Murine Leukemia Virus Reverse Transcriptase, and polyT primer as described previously (Chen et al. 2008). The gene specific primer pairs RubS-F (5-atggcttcctctatgctctcc-3) and RubS-R (5- gcaccggtgaagcttggtgg-3), and RubL-F (5-atgtcaccacaaacagagac-3) and RubL-R (5-gatctccttccatacttcac-3), were designed according the sequences of AtRubS and AtRubL and used for gene cloning. The gDNA and cDNA of TSS-AVRDC-2 and B-75 were treated as templates to amplify RubS and RubL sequences. The PCR products were purified by Qiagen gel extraction kits (Qiagen, Valencia, CA). The PCR products were ligated with a CloneJET PCR Cloning Kit (Thermo Scientific, Rochester, USA) using T4 DNA ligase. The inserts from individual colonies were amplified by PCR using the JetF (5-cgactcactatagggagagcgg-3) and JetR (5-aagaacatcgattttccatggcag-3) primers and then sequenced. DNA sequences were aligned and compared with reference sequences using ClustalW (http://www.ebi.ac.uk/Tools/msa/clustalw2).

### Northern blot analysis

Total RNA samples were extracted from plant leaves under stressed treatments to determine the expression levels of RubL and RubS in leaves of TSS-AVRDC-2 and B-75. Total RNA samples were separated by formaldehyde gel and blotted to Hybond N+ membranes (GE Healthcare, NJ, USA) using a trans-blot semidry transfer cell (Bio-Rad) for 1 h at 400 mA, followed by UV cross-linking. The radiolabeled RubL and RubS probes were produced by PCR reactions using TSS-AVRDC-2 cDNA clones as templates in the presence of α-^32^P-dATP with RubL-F and -R and RubS-F and RubS-R primers, respectively. PCR reactions were labeled with α-^32^P-ATP using High Fidelity *Taq* DNA Polymerase (Thermo Scientific). Blots used for the analysis were prehybridized with hybridized buffer (6X SSC, 5X Denhardt’s Solution, and 0.5 % sodium dodecyl sulfate) at 55 °C for 2 h. After the radiolabeled probes were added, hybridization was performed under the same conditions for another 16 h. Blots were washed twice in half-diluted saline-sodium citrate buffer and 0.1 % sodium dodecyl sulfate for 10 min, followed by washing once in 10 % saline-sodium citrate and 0.1 % sodium dodecyl sulfate for 15 min. Radioactive blots were then displayed on a Phosphor Imager (Molecular Dynamics, CA, USA). Ribosomal (r) RNA was analyzed after ethidium bromide staining and treated as a load control. The ratios of RubL and RubS RNA to 28S rRNA in each reaction were calculated and the ratio of the control reaction was treated as a value of one for determining the relative ratios of other reactions. To quantify expression levels, the radioactive signals and amounts of 28S rRNA were measured using computing laser densitometer ImageJ software (http://imagej.nih.gov/ij/), with radioactive signals being normalized by the amount of 28S rRNA. The expression level of treatment C was treated as 1 for normalizing the relative expression levels of H, F, and HF treatments. Each experiment was replicated three times.

## Results and discussions

### Phenotypic and physiological characteristics of broccoli plants treated by stresses

Plants display complex phenotypic and physiological responses to heat and flood stresses. It is necessary to identify physiological characteristics that reflect their complex underlying genetic make-up. Physiological phenotypes of 40-day-old TSS-AVRDC-2 and B-75 plants under control (C) and various stress treatments (F, H, and HF) for 12, 24, 48, and 72 h were observed. Leaf chlorophyll content (CC) is an indicator of photosynthetic efficiency when plant responding to stresses (Augustine et al. 2015). Significant changes in CC (measured as SPAD) were observed between and among genotypes under stress. Figure 1 shows that the SPAD values of both cultivars under C and F conditions were higher than those under H and HF conditions after 12 h. A significant reduction in SPAD values were observed in both cultivars treated by HF over time, but the reduction rate in B75 was more rapid than in TSS-AVRDC-2, indicating that the CC of both cultivars were not influenced by waterlogging and that TSS-AVRDC-2 maintained higher CC values than B-75 under high temperature and waterlogging treatments, except for after 24 h. The SC parameter was chosen as an indirect proxy of plant physiology under stress regarding the level of leaf water loss and CO_2_ uptake. SC values of B-75 plants under condition H from 24 h to120 h were significantly higher than in TSS-AVRDC-2 plants (Fig. 2), indicating that transpiration in B-75 was increased due to stomata being open at high temperatures. Moreover, the SC values of both cultivars under treatment H after 72 h were higher than in other treatments. No significant differences in SC values were observed between F and HF in both cultivars after 72 h. These results suggest that stomata were closed when plants were waterlogged and that stomata closure under waterlogging and high temperatures was faster than under either waterlogging or high temperature treatments alone. Excess accumulation of H_2_O_2_ can lead to oxidative stress in plants, which then triggers cell death. HC elevates under waterlogging and high temperatures stresses in plants (Simova-Stoilova et al. 2012; Xu et al. 2014). Therefore, HC was assessed over time under various treatments (Fig. 3). Compared to B-75 plants, TSS-AVRDC-2 plants had lower HC under H treatment from 24 to 48 h. Furthermore, the endogenous H_2_O_2_ content was elevated when both cultivars were exposed to HF stresses. Thus, high temperature plus waterlogging caused an overproduction of H_2_O_2_ in both genotypes, their H_2_O_2_ scavenging systems being unable to eliminate the extra H_2_O_2_. A combination of waterlogging and high temperature induced larger amounts of H_2_O_2_ than individual stresses in both cultivars.

Figures 1, 2 and 3 show that two cultivars displayed different physiological responses to various stress treatments. TSS-AVRDC-2 maintained significantly higher chlorophyll levels than B-75 under high temperature treatments at 24 h (Fig. 1), indicating that TSS-AVRDC-2 has a better photosynthetic efficiency than B-75, displaying its characteristically high temperature tolerance. Typically, CC is reduced in amount by stressful conditions (Xu et al. 2014). This is consistent with our observations that chlorophyll loss was more pronounced in heat-sensitive B75 plants in accordance with the more pronounced and increased visible symptoms of leaf injury. In addition, the SC values of B-75 were largely increased from 0 h to 72 h under high temperature, whereas TSS-AVRDC-2 exhibited low SC values in order to prevent water loss (Fig. 2). Although wilted leaves were observed in TSS-AVRDC-2 and B-75 under stress treatments for at least 24 h, SC values in both genotypes under F and HF treatment over time were low (Fig. 2). These results suggest that waterlogging results in stomata closure, which prevents transpiration. H_2_O_2_ acts as a ubiquitous marker of signaling events for the induction of adaptive stress responses and oxidative stress (Mittler et al. 2011). This dual function should be tightly regulated by the balancing of H_2_O_2_ production and scavenging (Mittler et al. 2004). When plants were treated with H_2_O_2_, their tolerances to high temperature and waterlogging were increased (Uchida et al. 2002; Banti et al. 2010). Plants often suffer from a simultaneous combination of high temperature and waterlogging. In our study, TSS-AVRDC-2 had a better H_2_O_2_ control system than B-75, and low levels of H_2_O_2_ increased tolerance to high temperature stress in TSS-AVRDC-2 (Fig. 3). However, waterlogging plus high temperature damaged broccoli plants more seriously than either high temperature or waterlogging alone, through the overproduction of H_2_O_2_. At the physiological level, the many effects of flooding and heat stresses indicate the importance of protecting plants from oxidative damage caused by the overproduction of H_2_O_2_.

### Differentially expressed proteins in plant leaves among stress treatments

Changes in protein accumulation under stress likely are directly related to the physiological phenotypic response of plants to stress. To investigate the effects of single and combined stress treatments on the differential expression of proteins, leaf total protein was extracted from stressed 40-day-old TSS-AVRDC-2 and B-75 plants at 12 h and 24 h. Fifteen and 16 protein peaks were identified from TSS-AVRDC-2 (Table 1; Additional file 1: Figure S1) and B-75 (Table 2; Additional file 2: Figure S2), respectively. These identified proteins emerged as key participants in stress tolerance. Among 31 identified proteins, the expressed levels of RubL and RubS in TSS-AVRDC-2 and B-75 plants under stress showed distinct differences. In TSS-AVRDC-2 plants, RubL protein (peak 5) was up-regulated under F treatment for 24 h compared to controls, and RubS protein (peak 13) was up-regulated under HF treatment for 24 h compared to controls (Table 3). Furthermore, two RubS proteins (peaks 24, 27) in B-75 were up-regulated by either for 12 h HF treatment or 24 h H treatment, while RubL proteins (peak 16) were down-regulated by F, H and HF treatments for 12 h (Table 4). Rubisco proteins are responsible for carbon fixation in plants and were regulated by stressing (Bose et al. 1999). To confirm these results, the RNA expression levels of RubL and RubS in TSS-AVRDC-2 and B-75 under stressing were analyzed.

**Table 1:** Identification of differentially expressed stress-related proteins in TSS-AVRDC-2 by MALDI-TOF-MS

Treatment	Stress Time (h)	Peak	Homologous protein	Species	PI	S^a^	Accession number
F	12	1	Putative F-box protein At5g41500	*Arabidopsis thaliana*	8.9	74	FB277_ARATH
	24	2	DNA-directed RNA polymerase subunit beta	*Geobacillus thermodenitrificans*	8.9	86	RPOC_GEOTN
		3	Conserved oligomeric Golgi complex subunit	*Magnaporthe grisea*	4.9	66	COG6_MAGO7
		4	ATP synthase subunit beta	*Brimeura amethystina*	5.2	76	ATPB_BRIAM
		5	Rubisco large chain	*Brassica oleracea*	5.8	96	RBL_BRAOL
		6	Mitochondrial 2-oxoglutarate/malate carrier protein	*Rattus norvegicus*	9.8	76	M2OM_RAT
H	24	7	ATP synthase subunit beta	*Nephroselmis olivacea*	5.2	90	ATPB-NEPOL
		8	Phosphopantetheine adenylyltransferase	*Silicibacter sp.*	5.4	76	EEW57108
		9	Ribosome-binding factor A	*Salmonella choleraesuis*	5.9	62	RBFA_SALCH
		10	Pentatricopeptide repeat-containing protein At2g01740	*Arabidopsis thaliana*	6.0	65	PP141_ARATH
HF	12	11	Putative F-box protein At5g41500	*Arabidopsis thaliana*	8.9	74	FB277_ARATH
		12	Eukaryotic translation initiation factor 3	*Saccharomyces cerevisiae*	5.4	54	EIF3I YEAST
	24	13	Rubisco small chain	*Brassica napus*	8.2	88	PO5346
		14	Ribosome-binding factor A	*Salmonella choleraesuis*	5.9	54	RBFA_SALCH
		15	Pentatricopeptide repeat containing protein At2g01740	*Arabidopsis thaliana*	6.0	65	PP141_ARATH

^a^: Probability-based molecular weight search (Mowse) score

F, waterlogging; H, 40 °C; HF, waterlogging at 40 °C

**Table 2:** Identification of differentially expressed stress-related proteins in B-75 by MALDI-TOF-MS

Treatment	Stressed Time (h)	Peak	Homologous protein	Species	PI	S^a^	Accession number
C	12	16	Rubisco large chain	*Brassica oleracea*	5.8	78	RBL_BRAOL
F	12	17	ATP synthase subunit beta	*Acutodesmus obliquus*	5.2	66	ATPB ACUOB
	24	18	Inorganic pyrophosphatase	*Corynebacterium efficiens*	4.5	70	Q8FMF8
		19	tRNA pseudouridine synthase B	*Mycobacterium avium*	8.2	74	A0QIX1
H	12	20	DNA-directed RNA polymerase subunit beta	*Staphylococcus haemolyticus*	6.1	61	RPOC_STAHJ
		21	Probable O-sialoglycoprotein endopeptidase	*Leptospira borgpetersenii Serovar hardjo-bovis*	5.7	75	ABJ76496
		22	Cysteinyl-tRNA synthetase	*Lactococcus lactis subsp.*	4.8	72	SYC_LACLS
	24	23	Formin like protein 19	*Arabidopsis thaliana*	9.1	56	Q9FF14
		24	Rubisco small chain	*Brassica napus*	8.2	98	PO5346
		25	Putative uncharacterized protein yibJ	*Escherichia coli*	5	60	P32109
		26	Thiamine biosynthesis protein thiC	*Shewanella halifaxensis*	5.1	78	B0TRP7
HF	12	27	Rubisco small chain	*Brassica napus*	8.2	97	RBS1_BRANA
		28	tRNA 2-thiocytidine biosynthesis protein ttcA	*Sorangium cellulosum*	7.0	76	TTCA_SORC5
		29	Myosin-1	*Canis familiaris*	5.6	83	MYH1_CANFA
	24	30	Putative uncharacterized protein yibJ	*Escherichia coli*	5	68	P32109
		31	Haptoglobin	*Mesocricetus auratus*	5.7	81	O35086

^a^Probability-based molecular weight search (Mowse) score

F, waterlogging; H, 40 °C; HF, waterlogging at 40 °C

**Table 3:** Highly differentially expressed protein pattern in stressed TSS-AVRDC-2

Stressed time (h)	Homologue protein	Flooding	40 °C	Flooding at 40 °C
12	Putative F-box protein At5g41500	+	-	+
	Eukaryotic translation initiation factor 3	-	-	+
24	DNA-directed RNA polymerase subunit beta	+	-	-
	Rubisco large chain	+	-	-
	Rubisco small chain	-	-	+
	Conserved oligomeric Golgi complex subunit	+	-	-
	ATP synthase subunit beta	+	+	-
	Mitochondrial 2-oxoglutarate/malate carrier protein	+	-	-
	Phosphopantetheine adenylyltransferase	-	+	-
	Ribosome-binding factor A	-	+	+
	Pentatricopeptide repeat-containing protein	-	+	+

+, up-regulated protein; −, down-regulated protein

**Table 4:** Highly differentially expressed protein pattern in stressed B-75

Stressed time (h)	Homologue protein	Flooding	40 °C	Flooding at 40 °C
12	Rubisco large chain	-	-	-
	Rubisco small chain	-	-	+
	ATP synthase subunit beta	+	-	-
	DNA-directed RNA polymerase subunit beta	-	+	-
	Probable O-sialoglycoprotein endopeptidase	-	+	-
	Cysteinyl-tRNA synthetase	-	+	-
	tRNA 2-thiocytidine biosynthesis protein ttcA	-	-	+
	Myosin-1	-	-	+
24	Rubisco small chain	-	+	-
	Inorganic pyrophosphatase	+	-	-
	tRNA pseudouridine synthase B	+	-	-
	Formin like protein 19	-	+	-
	Putative uncharacterized protein yibJ	-	+	+
	Thiamine biosynthesis protein thiC	-	+	-
	Haptoglobin	-	-	+

+, up-regulated protein; −, down-regulated protein

In the present study, we analyzed physiological and proteomics data in different genotypes of broccoli under high-temperature and waterlogging stresses for different time periods. For survival, plants must respond to flood and/or heat stresses differently from the way they regulate protein expression during biochemical and physiological adaptations. Photosynthesis is one of the systems that is most sensitive to high-temperature stress (Salvucci 2008; Wang et al. 2015). Photosynthesis systems were protected from the damages caused by waterlogging and/or heat in TSS-AVRDC-2, which led to physiological phenomena such as high CC and low SC and HC in leaves. Chlorophyll functioning is not decreased in stressed thermo-tolerant plants (Li et al. 2003; Xu et al. 2014). A temperature of 40 °C reduces photosynthesis, which seems to change the enzymatic response of a CO_2_-fixing enzyme, Rubisco (Markus et al. 1981). Thus, RubL and RubS were selected to do the further tests.

### RubL and RubS transcripts were regulated when stressed

**Fig. 4:**
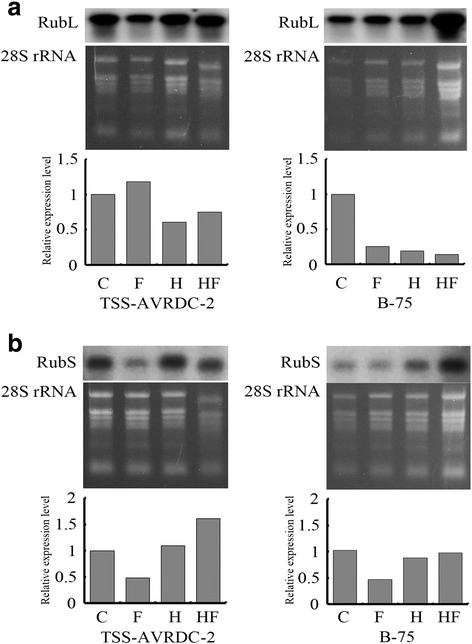
Expression levels of Rubisco genes in stressed plants. Expression analysis of leaf Rubisco large (RubL, **a**) and small (RubS, **b**) RNA in TSS-AVRDC-2 and B-75 plants under control (C), waterlogging (F), 40 °C (H), and waterlogging at 40 °C (HF) treatments for 72 h. Total RNA was analyzed by RNA gel blotting with cDNA sequences of RubL and RubS subunits as specific gene probes. Ethidium bromide staining of 28S rRNA gel was shown as a loading control. The ratios of RubL and RubS RNA to 28S rRNA in each reaction were calculated, and the ratio of the control reaction was treated as a value of one for determining the relative ratios of the other reactions

In our study, Rubisco subunits (small and large chain) related to photosynthesis were differentially expressed and regulated by combination treatments and between genotypes (Tables 1 and 2). RubL and RubS sequences were cloned from gDNA and cDNA of TSS-AVRDC-2 and B-75 using RubL-F and -R and RubS-F and -R in pairs. Cloned sequences were aligned and compared with *Arabidopsis* RubL and RubS genes. The sequences of RubL in gDNA were 97.4 % matched with reference sequences from *Arabidopsis* (Additional file 3: Figure S3), yet the RubS gene shows two introns in the gDNA sequences (Additional file 4: Figure S4). The cDNA identity of RubL and RubS between TSS-AVRDC-2 and B-75 was higher than 99 %. The transcript levels of RubL and RubS in TSS-AVRDC-2 and B-75 were analyzed by Northern blot. RubL and RubS transcripts in both genotypes under control treatment were observed (Fig. 4). Compared to the transcript level of RubL in TSS-AVRDC-2 controls at 72 h, RubL transcripts were increased by 18 % but decreased by 39 % and 25 % under F, H, and HF treatments, respectively (Fig. 4a). However, in B-75 plants, RubS transcripts decreased 75 %, 81 %, and 86 % at F, H and HF, respectively, compared to controls. RubS transcripts of TSS-AVRDC-2 decreased 51 % at condition F at 72 h, but increased by 10 % and 62 % under H and HF conditions, respectively, compared to controls (Fig. 4b). The transcript level of RubL in B-75 decreased 55 %, 14 %, and 5 % by F, H, and HF treatments at 72 h, respectively, compared to controls. These results suggest that the transcript abundance of RubL in TSS-AVRDC-2 was higher than that of B-75 under all stress treatments.

To increase energy metabolism, the expressions of proteins related to redox homeostasis and response to stimuli were up-regulated, thereby maintaining physiological balance during stress. An up-regulation in Rubisco levels may also indicate an increase in the photorespiration rate. Rubisco’s large and small subunits were both identified as accumulated proteins, and subsequently RubL and RubS genes were cloned from TSS-AVRDC-2 and B-75. The coding sequences of RubL and RubS between TSS-AVRDC-2 and B-75 were highly similar (Additional file 3: Figures S3 and Additional file 4: Figure S4), but their gene expression levels were different under stress treatments (Fig. 4). Compared to controls, the relative expression level of the RubL gene in TSS-AVRDC-2 plants under F stressing was 1.18, whereas the RubL transcript of the B-75 plant was 0.25 (Fig. 4a). However, the relative expression levels of the RubS gene did not change too much in either TSS-AVRDC-2 (0.49) or B-75 (0.45) under F stressing (Fig. 4b). A similar trend applied to the high temperature condition. The relative expression levels of the RubS gene did not change too much in either TSS-AVRDC-2 (1.1) or B-75 (0.86) under H stressing (Fig. 4b). However, the relative expression level of the RubL gene in B-75 under H stressing was 0.19, whereas the RubL transcript of TSS-AVRDC-2 only dropped to 0.61 compared to controls (Fig. 4a). These results indicate that TSS-AVRDC-2 is tolerant to high temperature in summer via the transcriptional control of RubL, and that the maintenance of RubL transcripts may allow for a better photosynthetic efficiency in its tolerance to high temperature. Rubisco is the main enzyme responsible for the carboxylation reaction in plants. High temperatures decrease the stability and also depress the carboxylation reaction rate of Rubisco. Miller et al. (2013) demonstrated that the thermo-tolerant *Synechococcus* lineage containing higher Rubisco stability than other lineages. The Rubisco large subunit content, Rubisco activity, and CO_2_ assimilation capacity were protected from high temperature stress damage by chloroplast-targeted DnaJ (CDJ) in tomato (Wang et al. 2015). The CDJ overexpressed plants exhibited higher chlorophyll contents and fresh weights than the CJD knock-down plants. Meanwhile, heat-tolerant rice cultivar, N 22, had higher thermostability of the Rubisco protein than heat-sensitive cultivar, IR 8, under high temperature (Bose et al. 1999). These results indicate that heat-tolerant cultivar has a protective mechanism against thermal degradation of Rubisco. The maintenance of higher Rubisco subunit protein may help plants survival from the heat damages.

In this study, distinct protein profiles of TSS-AVRDC-2 and B-75 were generated and used for identifying differentially expressed protein profiles. Some of the proteins identified have been well characterized in terms of their response to stresses, while others have not been well studied with respect to their roles in the stress responses of plants. A ribosomal-binding factor A family *HrpA* was found in heat treated *Mycobacterium bovis* Bacillus Calmette-Gue’rin (Ohara et al. 1997; Tabira et al. 2000). Also, arabidopsis ribosome-binding factor A homolog *RBF1* is also essential for plastid protein biosynthesis (Fristedt et al. 2014). The increasing of ribosomal-binding factor may help in the translation of proteins, and further help plants tolerate heat stress. Our results show that the abundance of ribosome-binding factor A (peaks 9 and 14) in TSS-AVRDC-2 plants was also up-regulated after 24 h of H and HF treatment as compared to controls. Protein synthesis pathways were up-regulated in TSS-AVRDC-2 and helped TSS-AVRDC-2 tolerate high temperatures. Taken together, the genetic difference was supported by the high number of proteins differentially regulated between genotypes. Our data suggest that the early response of broccoli to flooding and high temperature might be an important stress adaptation for survival following not only hypoxia and heat, but also direct damage to cells by flooding and high temperature. All of the identified proteins likely work cooperatively to reestablish cellular homeostasis under stress. These findings are important for farming in high temperature areas and wetlands or other areas subject to short and intense rainfall events.

## Conclusions

Environmental stresses represent the most limiting conditions for horticultural productivity and plant exploitation worldwide. The long term goal of our work is to help breed a competitively higher flood- and heat-tolerant broccoli to be grown in lowlands during summer. The identification of the unique stress-responsive proteins from TSS-AVRDC-2 under stressing, such as RubL, RubS, and ribosome- binding factor A, will allow further dissection of the genetic basis of this transgressive performance in its offspring. Our results not only provide information for selecting lines having better tolerance to waterlogging and heat stresses, but also provide a basis for understanding broccoli metabolic pathways and their cross-talk under stress.

## Additional files

Additional file 1: Figure S1. Representatives of second-column separation in TSS-AVRDC-2 fractions. Leaf proteins from TSS-AVRDC-2 plants treated with 22 °C (C; red lines), flooding at 22 °C (F), 40 °C (H), and waterlogging at 40 °C (FH) for 12 and 24 h were separated with a two-dimensional protein fractionation (PF2D) system. The Y-axis represents the optical densitometry pattern (OD at 214 nm). The X-axis represents retention time in minutes according to the concentration of acetonitrile in the mobile phase. Red lines indicate the absorbance after treatment C. Green lines indicate the absorbance from treatment F (a to d), treatment H (e to g), and treatment HF (h to l), and the detected peaks were designated by numbers.

Additional file 2: Figure S2. Representatives of the second column separation of B-75 fractions. Leaf proteins from B-75 plants treated with 22 °C (C; red lines), flooding at 22 °C (F), 38 °C (H), and waterlogging at 40 °C (FH) for 12 and 24 h were separated with PF2D. The Y-axis represents optical densitometry pattern (OD at 214 nm). The X-axis represents retention time in minutes according to the concentration of acetonitrile in the mobile phase. Red lines indicate the absorbance from treatment C. Green lines indicate the absorbance from treatment F (b and d), treatment H (a and e to j), and treatment HF (k to o), and the detected peaks were designated by numbers.

Additional file 3: Figure S3. RubL DNA sequence comparison analyses by ClustalW. cDNA sequences of RubL from TSS-AVRDC-2 and B-75 were compared with RubL in *Arabidopsis*, including AtRubL (ATCG00490). ‘Star’ indicates identical residues in all sequences. The line above the ATG letters indicates the translation start site.

Additional file 4: Figure S4. RubS DNA sequence comparison analyses by ClustalW. cDNA sequences of RubS from TSS-AVRDC-2 and B-75 were compared with RubS in *Arabidopsis*, including AtRubS (AT5G38420). ‘Star’ indicates identical residues in all sequences. The line above ATG letters indicates the translation start site.

## Abbreviations

CC: Chlorophyll content
H_2_O_2_: Hydrogen peroxide
MALDI-TOF-MS: Matrix-assisted laser desorption ionization time-of-flight mass spectrometry
PF2D: Two-dimensional protein fractionation
RubL: Ribulose bisphosphate carboxylase large chain
RubS: Ribulose bisphosphate carboxylase small chain
SC: Stomatal conductance
