# Supplementary figures and images for: Proteomic analysis of broccoli (Brassica oleracea) under high temperature and waterlogging stresses
Source: Bot Stud. 2015 Jul 15;56:18. doi: 10.1186/s40529-015-0098-2 (PMC5432913; doi:10.1186/s40529-015-0098-2)

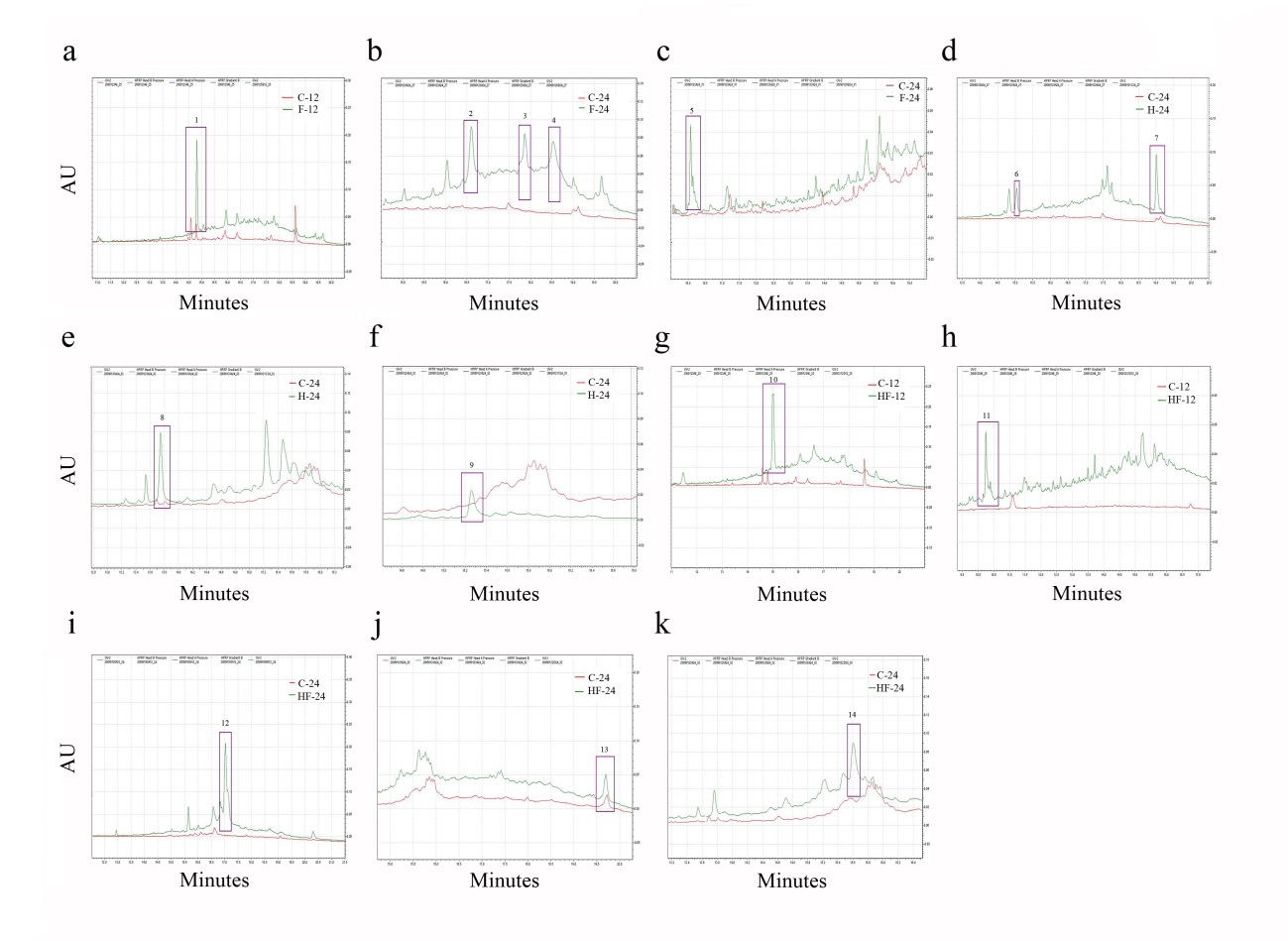


**Additional Figure S1**

Supplement: Supplementary file 1 — Representatives of second-column separation in TSS-AVRDC-2 fractions. Leaf proteins from TSS-AVRDC-2 plants treated with 22 °C (C; red lines), flooding at 22 °C (F), 40 °C (H), and waterlogging at 40 °C (FH) for 12 and 24 h were separated with a two-dimensional protein fractionation (PF2D) system. The Y-axis represents the optical densitometry pattern (OD at 214 nm). The X-axis represents retention time in minutes according to the concentration of acetonitrile in the mobile phase. Red lines indicate the absorbance after treatment C. Green lines indicate the absorbance from treatment F (a to d), treatment H (e to g), and treatment HF (h to l), and the detected peaks were designated by numbers. [file 40529_2015_98_MOESM1_ESM.docx]

**
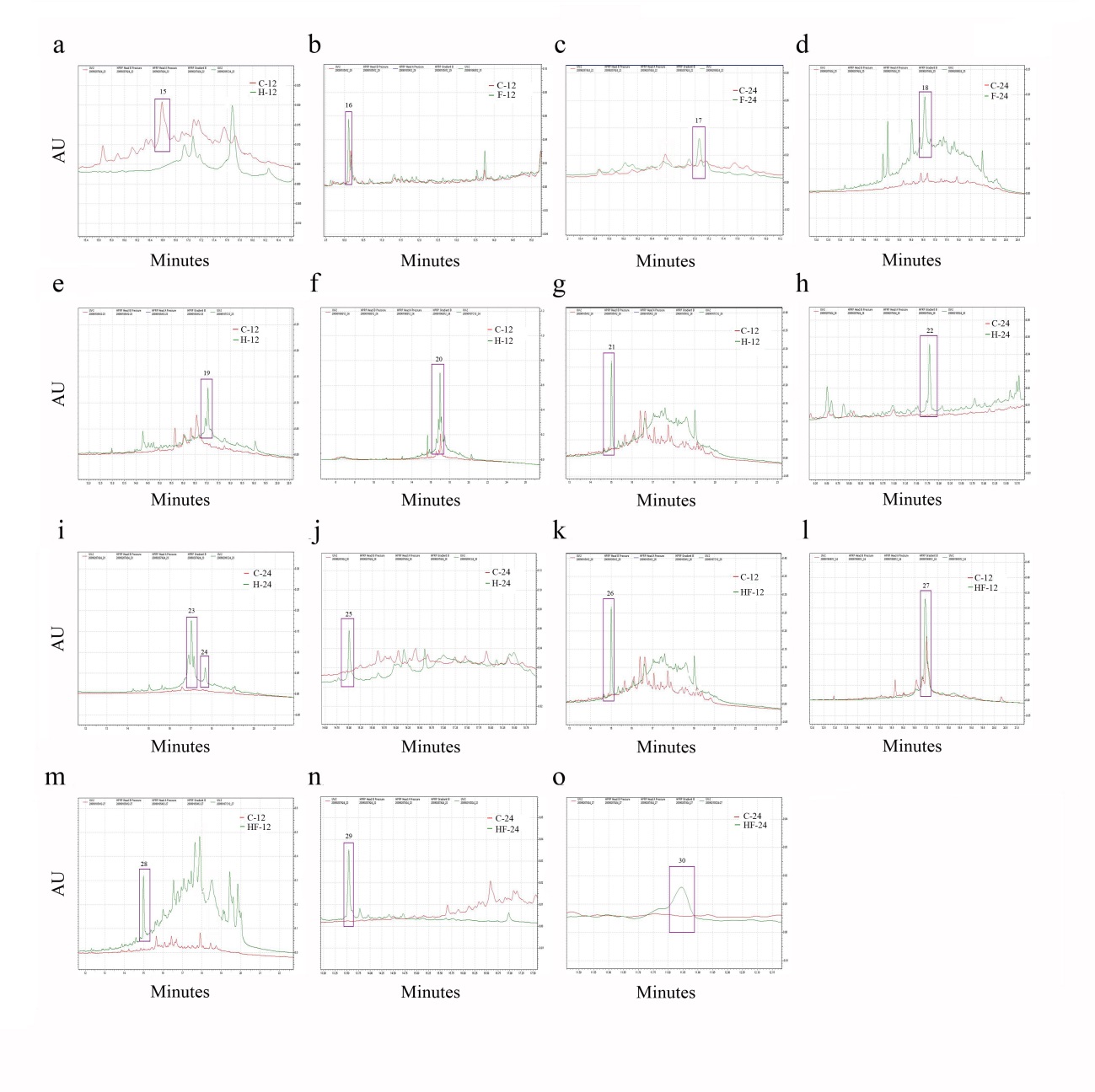
**

**Additional Figure S2**

Supplement: Supplementary file 2 — Representatives of the second column separation of B-75 fractions. Leaf proteins from B-75 plants treated with 22 °C (C; red lines), flooding at 22 °C (F), 38 °C (H), and waterlogging at 40 °C (FH) for 12 and 24 h were separated with PF2D. The Y-axis represents optical densitometry pattern (OD at 214 nm). The X-axis represents retention time in minutes according to the concentration of acetonitrile in the mobile phase. Red lines indicate the absorbance from treatment C. Green lines indicate the absorbance from treatment F (b and d), treatment H (a and e to j), and treatment HF (k to o), and the detected peaks were designated by numbers. [file 40529_2015_98_MOESM2_ESM.docx]

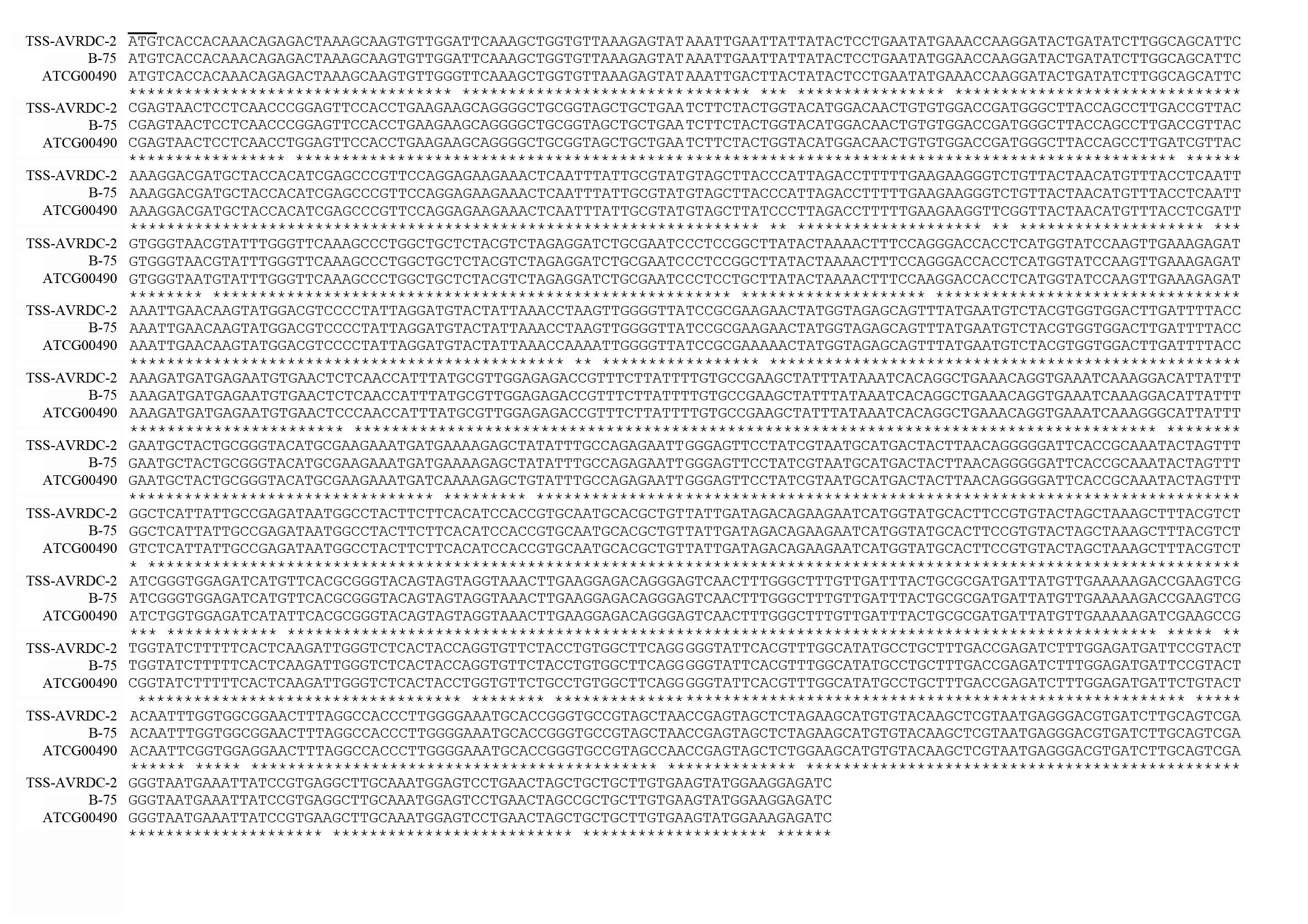


**Additional Figure S3**

Supplement: Supplementary file 3 — RubL DNA sequence comparison analyses by ClustalW. cDNA sequences of RubL from TSS-AVRDC-2 and B-75 were compared with RubL in Arabidopsis, including AtRubL (ATCG00490). ‘Star’ indicates identical residues in all sequences. The line above the ATG letters indicates the translation start site. [file 40529_2015_98_MOESM3_ESM.docx]

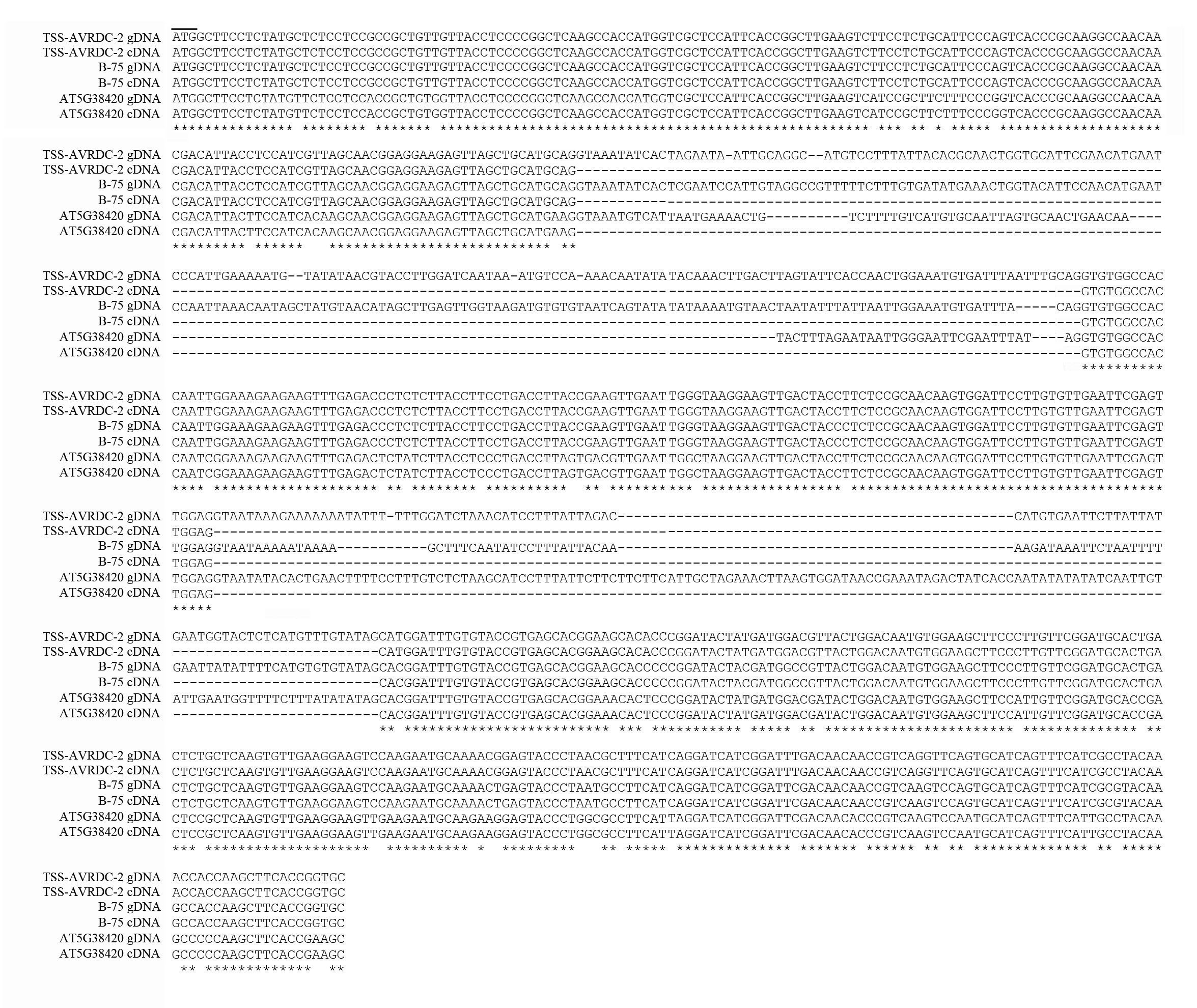


**Additional Figure S4**

Supplement: Supplementary file 4 — RubS DNA sequence comparison analyses by ClustalW. cDNA sequences of RubS from TSS-AVRDC-2 and B-75 were compared with RubS in Arabidopsis, including AtRubS (AT5G38420). ‘Star’ indicates identical residues in all sequences. The line above ATG letters indicates the translation start site. [file 40529_2015_98_MOESM4_ESM.docx]
